# Association Between Quality of Discharge Teaching and Post-Discharge Coping Difficulty in Postoperative Lung Cancer Patients: A Chain Mediation Model

**DOI:** 10.3390/curroncol32080468

**Published:** 2025-08-18

**Authors:** Minghui Wang, Hailing Tu, Jingfang Hong

**Affiliations:** School of Nursing, Anhui Medical University, Hefei 230032, China

**Keywords:** lung cancer, postoperative, discharge, coping, teaching, self-efficacy, readiness, mediation analyses

## Abstract

The post-discharge coping difficulties experienced by patients can affect their quality of life and the occurrence of unplanned readmissions. This study aimed to explore the chain mediation effect of self-efficacy and readiness for hospital discharge between quality of discharge teaching and post-discharge coping difficulty among postoperative lung cancer patients. This study employed a cross-sectional design and surveyed 358 postoperative patients with lung cancer. Demographic and Disease-Related Data Questionnaire, Quality of Discharge Teaching Scale, General Self-Efficacy Scale, Readiness for Hospital Discharge Scale, and Post-Discharge Coping Difficulty Scale were used. A structural equation model was utilized to explore the mediation effects of self-efficacy and readiness for hospital discharge. The total score for post-discharge coping difficulty among postoperative lung cancer patients was 34.32 ± 10.00. Quality of discharge teaching not only directly negatively predicted post-discharge coping difficulty (β = −0.154, *p* < 0.05), but also indirectly affected it through the chain mediation effect of self-efficacy and readiness for hospital discharge (β = −0.040, *p* = 0.001). Healthcare providers should pay attention to postoperative lung cancer patients’ post-discharge coping difficulties and formulate targeted discharge teaching strategies to enhance patients’ self-efficacy and readiness for discharge to alleviate their post-discharge coping difficulties.

## Simple Summary

Postoperative lung cancer patients face challenges affecting their quality of life after discharge. Although evidence suggests that high-quality discharge teaching can reduce such challenges, the mechanism remains unclear in postoperative patients with lung cancer. This study aims to explore the impact of the quality of discharge teaching on post-discharge coping difficulties faced by patients who have undergone lung cancer surgery and to reveal the mediating role of self-efficacy and readiness for discharge in these two factors. This study surveyed 358 postoperative lung cancer patients. Results show that postoperative lung cancer patients experience moderate post-discharge coping difficulties. Significantly, high-quality discharge teaching not only directly alleviates post-discharge coping difficulties in patients after lung cancer surgery but also indirectly improves this situation by enhancing their self-efficacy and readiness for hospital discharge. This finding provides an important basis for the development of targeted intervention strategies.

## 1. Introduction

According to statistics from the National Cancer Registry of China in 2022, lung cancer accounted for the highest number of new cases (1,060,600) and deaths (733,300) among all malignant tumors nationwide [1]. The extensive use of low-dose spiral CT screening has greatly enhanced the detection rate of early-stage lung cancer [2], and surgery remains the primary treatment method for these patients [3]. Patients’ hospital stays have been significantly shortened with the rapid development of minimally invasive techniques and enhanced recovery after surgery. However, shorter hospital stays result in patients being discharged before their physical functions fully recover, which increases the risk of readmission and complications after discharge [4]. This means that post-discharge rehabilitation management for lung cancer surgery patients is highly challenging. Therefore, it is essential to understand the elements that can contribute to patients’ successful transition from the hospital to their home. It may be an important breakthrough in enhancing postoperative rehabilitation management for lung cancer and improving patient prognosis.

Post-discharge coping difficulty (PDCD) refers to the challenges patients encounter after leaving the hospital, including life stress, physical recovery, self-care, wound care, and psychological, social, and medical information. It is among the frequently used indicators of post-discharge outcomes [5]. Research shows that systematic assessment of post-discharge coping difficulties is essential for improving patients’ quality of life, reducing unplanned readmission rates, and optimizing the allocation of healthcare resources [6]. According to recent research, patients undergoing lung cancer surgery experience symptoms such as shortness of breath, pain, and coughing after hospital discharge. These symptoms can last for a long time and have significant negative impacts on the patients’ psychological state and quality of life [7]. Unplanned readmissions following lung cancer resection are common and are associated with a six-fold increase in 90-day mortality [8]. In addition, the recurrence rate after early-stage non-small-cell lung cancer surgery is high, ranging from 30% to 55% according to reports [9]. Accordingly, exploring strategies that help improve patients’ ability to cope with challenges after hospital discharge is necessary to improve their prognosis and quality of life.

Quality of discharge teaching (QDT) involves the information and support that healthcare professionals offer patients while hospitalized. Patients who benefit from effective discharge education are better equipped to handle challenges after leaving the hospital [10]. A growing body of empirical research has demonstrated that patients’ self-management skills and post-discharge outcomes are significantly influenced by the quality of discharge teaching [4,11]. The guidance on the hospital-to-home transition is linked to the occurrence of adverse events, such as medication errors, problems with follow-up appointments, worsening symptoms, and patients’ lack of preparedness for self-management at home [12]. However, the connection between the quality of discharge teaching provided to lung cancer patients after surgery and their subsequent coping difficulties post-discharge is still ambiguous.

Self-efficacy (SE) is an individual’s expectations, confidence, cognitive beliefs, and convictions about their ability to take necessary actions to achieve specific goals effectively. These expectations affect how coping behaviors initiate, the effort exerted, and the duration of persistence when confronting challenges and discomfort experiences [13]. Consequently, cancer patients who have high expectations of self-efficacy are better at coping by efficiently using their skills and rallying their efforts. According to an empirical study [14], self-efficacy among mothers of premature babies is negatively correlated with their ability to cope with difficulties post-discharge. Mothers with low self-efficacy lack confidence in their parenting skills and have difficulty coping effectively with challenges, which exacerbates the difficulties they face. However, the mechanism between self-efficacy and post-discharge coping difficulties in patients after lung cancer surgery needs further empirical support.

Readiness for hospital discharge (RHD) is a comprehensive assessment of a patient’s health condition to determine if they can be discharged or need to continue rehabilitative care. It is a predictive indicator of whether a patient can effectively recover following discharge from the hospital [15]. A well-prepared discharge lowers medical expenses and improves outcomes after discharge [4]. Discharge readiness is positively impacted by self-efficacy [14]. Prior research has demonstrated that better discharge education is linked to increased patient self-efficacy and release readiness [11]. In postoperative lung cancer patients, however, the mechanisms of interaction between the quality of discharge teaching, self-efficacy, readiness for hospital discharge, and post-discharge coping difficulties have not been thoroughly examined.

**Figure 1:**
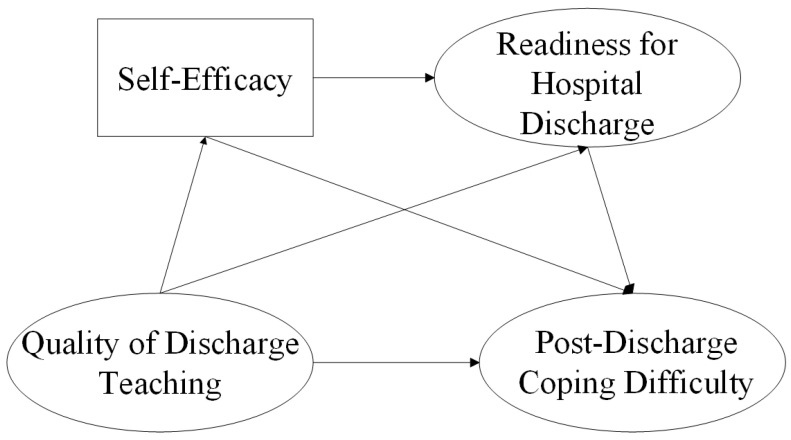
The hypothetical model of post-discharge coping difficulty.

Meleis’s transition theory is widely used to guide discharge nursing studies, helping to conceptualize the discharge transition and identify relevant study variables. As per this theory, transition refers to the change from one state, condition, or location to another [16]. This study selected Meleis’s transition theory as its theoretical framework, as transition theory aligns well with post-discharge transitions back to family. Transition theory primarily encompasses four aspects: the nature of transition, transition conditions, patterns of response, and nursing therapeutics. The nature of transition, transition conditions, and response patterns are interrelated, influencing one another significantly. Nursing therapeutics impact these three components while receiving feedback [17]. Based on transition theory, the transition of postoperative lung cancer patients returning home after hospital discharge is a situational transition with multiple patterns. As a key event in the patient’s transition experience, the transition property of hospital discharge can be defined as a critical point and event. Demographic and disease-related information and self-efficacy of postoperative lung cancer patients were used to represent transition conditions. The nursing therapeutic is the quality of discharge teaching. The process indicator of the response pattern is the patient’s readiness for hospital discharge, and the outcome indicator is the patient’s post-discharge coping difficulty. Three hypotheses based on transition theory were proposed in this study (Figure 1). We aim to better understand the challenges postoperative lung cancer patients encounter after discharge by investigating the mechanisms underlying these variables’ interactions, which will offer valuable insights for clinical nursing practice.

**H1:**  *Quality of discharge teaching, self-efficacy, readiness for hospital discharge, and post-discharge coping difficulty are all correlated in postoperative lung cancer patients*.

**H2:**  *Self-efficacy and readiness for hospital discharge each act as mediators between the quality of discharge teaching and post-discharge coping difficulty in postoperative lung cancer patients*.

**H3:**  *Between the quality of discharge teaching and post-discharge coping difficulty in postoperative lung cancer patients, self-efficacy and readiness for hospital discharge have a chain mediating role*.

## 2. Methods

### 2.1. Study Design and Participants

This is a cross-sectional study that followed the Strengthening the Reporting of Observational Studies in Epidemiology (STROBE) guidelines (Supplementary Materials) [18]. It was conducted from September 2024 to April 2025 in the general thoracic surgery department of a tertiary hospital in Hefei, China. Participants were selected using convenience sampling, and the following criteria were used to determine which patients were included: (1) non-small-cell lung cancer (NSCLC) with pathological diagnosis [19]; (2) age ≥ 18 years; (3) patients who voluntarily cooperated with the participation in this study and were able to fill in the content in writing or answer orally; (4) patients undergoing radical surgery for lung cancer for the first time and who have been approved for hospital discharge. The following were the criteria for exclusion: (1) those who had communication difficulties with hearing dysfunction; (2) patients with a history of primary psychiatric disease; and (3) those who could not cooperate with text comprehension disorders or miscommunication.

According to the sample size requirements for structural equation model (SEM), a baseline sample size of 200 participants was established, with an additional 5 to 10 participants required per added variable [20]. This study included nine observed variables, resulting in a calculated maximum sample size of 290 participants. Considering that invalid questionnaires or other error factors may have interfered with the sample recovery process, the sample size was increased by 10%. Consequently, 323 was the exact sample size required.

### 2.2. Measurements and Instruments

#### 2.2.1. Demographic and Disease-Related Data Questionnaire

This questionnaire was designed based on a combination of literature review and research objectives, including (1) demographic data like sex, age, marital status, educational degree, employment, etc., and (2) disease-related information, such as postoperative length of stay, pain score at discharge, TNM staging, and occurrence of pre-discharge complications.

#### 2.2.2. Post-Discharge Coping Difficulty Scale

Developed by Fitzgerald Miller et al. [5], this instrument is administered via telephone follow-up three weeks after discharge. The Chinese version of Xueqin Zhao’s Post-Discharge Coping Difficulty Scale (PDCDS) was employed in this study. It consists of seven items with two dimensions (living management and emotional management), and each item is given a score between 0 and 10. Higher total scores suggest that patients have greater difficulty coping with their difficulties after discharge. With a Cronbach’s α of 0.892, a retest reliability of 0.951, and a CVI of 0.94, the Chinese version of the PDCDS has demonstrated good validity and reliability [21]. In this study, the scale’s Cronbach’s α was 0.931.

#### 2.2.3. Quality of Discharge Teaching Scale

Weiss et al. created the scale [22]. The Quality of Discharge Teaching Scale (QDTS), translated by Binghua Wang, was utilized in this study. The scale encompasses three dimensions, totaling 24 items: patients’ self-perceived pre-discharge needs (6 items), actual support received (6 items), and effectiveness and technique of discharge teaching (12 items). A 0–10 point scale was used to rate the scale items, with higher overall scores denoting stronger teaching quality. The instrument has good reliability (Cronbach’s α of 0.924) and overall validity (0.98) [23]. This study’s scale had a Cronbach’s α of 0.928.

#### 2.2.4. General Self-Efficacy Scale

We made use of the Chinese version of the General Self-Efficacy Scale (GSES), which was initially established by Ralf Schwarzer et al. [24] and translated into Chinese by Wang Caikang et al. Since then, it has been widely used in Chinese research. The scale is unidimensional, with 10 items and a 0–4 point rating system. Higher total scores indicate stronger overall self-assurance when facing diverse challenges. For the GSES in Chinese, the Cronbach’s α is 0.87 [25]. The current study’s Cronbach’s α was 0.874.

#### 2.2.5. Readiness for Hospital Discharge Scale

The scale was initially proposed by American scholars Weiss et al. [26], and this study used a cross-cultural adaptation of the Readiness for Hospital Discharge Scale (RHDS) by Lin et al. Dimensions were divided into personal status (items 1 to 3), coping ability (items 4 to 8), and expected support (items 9 to 12), and every entry is rated on a scale from 0 to 10, where higher total points suggest higher readiness for discharge. Its Cronbach’s α is 0.89, and the content validity is 0.88 [27]. The Cronbach’s α in this research was 0.925.

### 2.3. Procedure

This study conducted two rounds of surveys. Before postoperative lung cancer patients were discharged from the hospital, a total of 380 questionnaires (QDTS, RHDS, and GSES) were distributed after the patients’ informed consent was obtained. The Demographic and Disease-Related Data Questionnaire was completed by researchers based on the patients’ electronic medical records. In the first round of surveys, a total of 370 valid questionnaires were received, with a valid recovery rate of 97.4% (370/380). In the second stage, when assessing patients’ PDCDS by telephone follow-up three weeks after hospital discharge, 12 cases were lost to follow-up (7 participants could not be contacted, and another 5 chose to withdraw). A total of 358 valid samples with complete two-stage data were ultimately obtained. Based on the initial distribution of 380 questionnaires, the overall valid response rate was 94.2% (358/380). Throughout the data collection procedure, the researchers confirmed the integrity of the data and offered standardized explanations.

### 2.4. Data Analysis

After collation, the data were imported into an Excel database and statistically analyzed using SPSS (Version 26.0) and AMOS (Version 26.0). Frequencies and percentages were used to represent categorical variables, while mean ± SD was used to express continuous variables with a roughly normal distribution. Independent-sample *t*-tests and a one-way ANOVA were conducted to examine PDCD-associated factors. Spearman’s rank correlation analysis assessed QDT, SE, RHD, and PDCD correlations. A structural equation model was utilized to identify the effects of QDT, SE, and RHD on patient PDCD and to analyze the mediation mechanisms. Maximum likelihood estimation was employed to estimate model parameters and obtain the best fit results. Meanwhile, the Bootstrap method was used to test the mediating effect of each variable further to verify the reliability and explanatory ability of the model.

## 3. Results

### 3.1. Participants’ Characteristics

Of the participants with postoperative lung cancer in this research, 184 (51.4%) were female and 174 (48.6%) were male; the average age was 57.22 ± 13.03 years; 86.6% of the patients were married; 70.9% of the patients were hospitalized for ≤5 days after the operation; and 48.0% of the patients had a disease stage of stage II. More information is shown in Table 1.

### 3.2. The Level of Post-Discharge Coping Difficulty

The total PDCDS score of postoperative lung cancer patients was 34.32 ± 10.00, with a mean item score of 4.90 ± 1.43, at the middle level. The total score of the emotional management dimension was 9.07 ± 2.98, the mean item score of 4.53 ± 1.49; the total score of the living management dimension was 25.25 ± 7.39, with a mean item score of 5.05 ± 1.48. See Table 2 for details of the mean scores of each item.

### 3.3. Correlation Analysis

The total QDTS score of the 358 postoperative lung cancer patients was 139.29 ± 21.45, which was at the middle level. The total GSES score was 24.39 ± 5.94, at the upper-middle level. The total RHDS score was 72.72 ± 13.12, at the middle level. The PDCDS scores showed negative correlations with QDTS scores (*r* = −0.447, *p* < 0.01), GSES scores (*r* = −0.575, *p* < 0.01), and RHDS scores (*r* = −0.522, *p* < 0.01). The correlations between the score of PDCDS and its dimensions with the QDTS, GSES, and RHDS scores in patients are presented in Table 3.

### 3.4. Structural Equation Model and Mediation Effect Analysis

**Figure 2:**
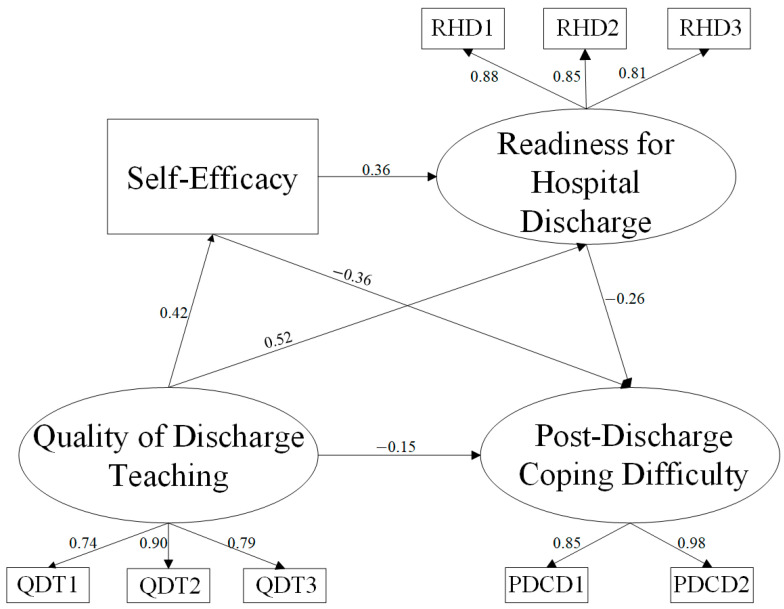
Model for the effect of quality of discharge teaching, self-efficacy, and readiness for hospital discharge on post-discharge coping difficulty. All path coefficients were standardized. Abbreviations: QDT1, patients’ self-perceived pre-discharge needs dimension; QDT2, actual support received dimension; QDT3, effectiveness and technique of discharge teaching dimension. RHD1, personal status dimension; RHD2, coping ability dimension; RHD3, expected support dimension. PDCD1, emotional management dimension; PDCD2, living management dimension.

This study investigates the impact pathways and effects of QDT, SE, and RHD on PDCD in postoperative lung cancer patients. The structural equation model examining PDCD influencing factors incorporated only these three variables. The measurement model demonstrated good fit indices (χ^2^/df = 2.968, NFI = 0.967, IFI = 0.978, CFI = 0.978, GFI = 0.963, AGFI = 0.923, RMSEA = 0.074) (Figure 2). All path coefficients were statistically significant. Specifically, QDTS, GSES, and RHDS directly influenced PDCDS: QDTS exhibited a direct negative predictive effect on PDCDS (*β* = −0.154, *p* < 0.05). QDTS positively predicted both GSES (*β* = 0.420, *p* < 0.001) and RHDS (*β* = 0.520, *p* < 0.001). GSES positively influenced RHDS (*β* = 0.361, *p* < 0.001) while negatively predicting PDCDS (*β* = −0.356, *p* = 0.001), and RHDS exhibited a negative correlation with PDCDS (*β* = −0.264, *p* < 0.05). The above results supported hypothesis H1. Mediation analyses revealed significant indirect effects: QDTS influenced PDCDS through GSES (*β* = −0.15, *p* < 0.001) and RHDS (*β* = −0.137, *p* = 0.001). Hypothesis H2 was also supported. Furthermore, a chain mediating effect involving GSES and RHDS was identified between QDTS and PDCDS (*β* = −0.040, *p* = 0.001), which supported hypothesis H3 (Table 4).

## 4. Discussion

The results demonstrate that postoperative lung cancer patients scored 34.32 ± 10.00 on the PDCDS, which was considered moderate. This level is comparable to that of postoperative esophageal cancer patients [15]. Collectively, these findings indicate the need for measures to mitigate the challenges faced by cancer patients in the post-discharge period. Postoperative lung cancer patients must cope with a heavy burden of symptoms [28], psychological stress due to disease ‘stigma’ [29], and complex postoperative management demands [30]. These factors together may lead to difficulties in coping post-discharge. Further analysis indicated that the mean item score for the living management dimension (5.05 ± 1.48) exceeded that of the emotional management dimension (4.53 ± 1.49). This suggests that patients with lung cancer after surgery encountered more significant challenges in coping post-discharge concerning self-care, family concerns, necessary assistance, emotional support, and healthcare management. This phenomenon may also be associated with the intricate postoperative care necessities of individuals diagnosed with lung cancer. The above findings highlight the urgent need to develop a targeted transitional care support system for patients who have undergone lung cancer surgery.

This study showed that post-discharge coping difficulties in patients after lung cancer surgery were influenced by age, marital status, educational degree, monthly household income, and TNM staging. Regarding age, the reason could be that cancer patients’ capacity to manage different stressors efficiently declines with age [31]. Marital status has a profound impact on patients’ health [32]. Postoperative lung cancer patients who have experienced divorce or widowhood may experience severe psychological stress and weakened social support, ultimately compromising health outcomes. Patients who are more educated generally have greater health awareness and demonstrate more effective health behaviors, which contribute to improved adaptation after discharge [33]. Low-income postoperative lung cancer patients experienced greater post-discharge coping difficulties compared to others. This aligns with previous findings [34]. Low-income postoperative lung cancer patients often find themselves in a difficult situation due to the high cost of medication, which may prevent them from receiving the most effective treatment in the initial stages of the disease, due to financial reasons. This can adversely affect their health outcomes. Many patients with higher TNM lung cancer require preoperative neoadjuvant therapy to improve surgical resectability due to significant tumor invasion. While potentially reducing tumor size, these treatments carry risks of adverse effects, such as pneumonia and gastrointestinal complications, which complicate postoperative recovery. Furthermore, the need for subsequent adjuvant therapy post-surgery leads to cumulative adverse effects, prolonging recovery and imposing substantial coping challenges [35]. To effectively address the coping challenges of these postoperative lung cancer patients, healthcare providers should create personalized discharge guidance programs tailored to individual characteristics. Additionally, they are encouraged to enhance multidisciplinary collaborative intervention mechanisms to optimize the rehabilitation outcomes.

The results of this study indicated a negative correlation between post-discharge coping difficulty and the quality of discharge teaching among patients who have undergone lung cancer surgery. Effective hospital discharge education can significantly reduce the challenges and problems faced by lung cancer patients undergoing postoperative rehabilitation at home by providing them with comprehensive professional knowledge and appropriate coping methods. This supports Meleis’s transition theory, which proposes that education is the primary strategy for assisting patients in making a smooth transition [17]. Therefore, effective interventions should be implemented to improve the quality of hospital discharge education for patients undergoing lung cancer surgery, thereby promoting their successful transition.

This study found that, among patients who underwent lung cancer surgery, the quality of discharge teaching indirectly affected post-discharge coping difficulties through the mediating effect of self-efficacy. Specifically, improving the quality of hospital discharge education for postoperative lung cancer patients can enhance their confidence and ability to cope with challenges, which can effectively reduce post-discharge difficulties. Previous research has also demonstrated that psychological therapeutics paired with precise health guidance can significantly enhance lung cancer patients’ cognitive functioning and subjective well-being. This approach not only reduces their anxiety and depression but also improves their overall quality of daily life [36]. Analyzing the reasons behind these findings, good discharge teaching content as an external factor provides patients with support from a medical perspective. In contrast, self-efficacy as a characteristic of the patients themselves further strengthens the effectiveness of discharge education on improving coping abilities.

Findings from this study suggest that readiness for hospital discharge among postoperative lung cancer patients mediates between the quality of discharge teaching and post-discharge coping difficulties. Postoperative lung cancer patients receiving enhanced instruction are better prepared for discharge, which boosts their ability to handle challenges after leaving the hospital. This conclusion aligns with the research by Zhang et al. [4], which suggests that practices and services led by nurses can improve patients’ preparation for discharge, thus impacting their post-discharge outcomes. Moreover, Meleis’s transition theory emphasizes that transitions occur as time passes [17]. Recognizing the process indicators that lead individuals toward either health or vulnerability and risk allows nurses to make timely assessments and interventions, ultimately fostering positive health outcomes. Therefore, improving the quality of discharge teaching for postoperative lung cancer patients and prioritizing patient preparation can help improve their discharge experience and support the recovery process.

It is worth noting that the quality of discharge teaching indirectly affects post-discharge coping difficulty through the chain mediation of self-efficacy and readiness for hospital discharge. Specifically, discharge education directly increases coping skills through self-efficacy of postoperative lung cancer patients and optimizes their hospital discharge preparedness by increasing self-efficacy, which lessens post-discharge coping challenges. A study of patients who underwent cataract surgery also confirmed that effective discharge teaching boosts patients’ adaptation to new environments, enhances confidence for transitioning home, and increases preparedness, while patient readiness before discharge lays the foundation for post-discharge health outcomes [4]. The findings of this study were consistent with the primary perspective of Meleis’s transition theory, which stated that conditions that prevent or promote transition, such as the nursing skills that patients master, have a certain degree of influence on patients’ coping patterns (health outcomes) [17]. Future researchers may consider this theoretical perspective, promoting high-quality discharge education, fostering self-efficacy, and ensuring comprehensive readiness, advancing these factors as interrelated interventions synergistically.

### 4.1. Clinical Implications

This study provides insights into post-discharge rehabilitation support for postoperative lung cancer patients. After lung cancer surgery, patient often have symptoms like persistent pain, fatigue, and shortness of breath, which can hurt their mental state and quality of life. The high recurrence risk adds uncertainty, increasing stress and making post-discharge coping more challenging. These linked factors indicate that healthcare professionals require proactive, careful support during this vulnerable post-discharge period for these patients. Drawing on this study’s findings, the first critical step is to implement evidence-based strategies for tailoring discharge teaching to the specific needs of lung cancer patients, which begins with a comprehensive assessment of individual differences. This assessment encompasses sociodemographic factors (e.g., age, marital status) and disease-related characteristics (e.g., TNM stage). This assessment informs the development of a high-quality discharge education program that combines structured and personalized elements. Structured components ensure the standardized delivery of essential information, including life stress management, physical rehabilitation guidance, self-care techniques, wound care procedures, and psychosocial/medical information. Meanwhile, personalized adjustments address unique needs, such as simplified guidance for older patients with lung cancer or targeted psychological counseling for those who are divorced or widowed. Beyond tailoring content, the study underscores the need for a proposed framework integrating self-efficacy and discharge readiness assessments into routine care; by systematically evaluating postoperative lung cancer patients’ confidence in managing daily challenges and their preparedness to transition home, nurses can refine educational interventions to strengthen these critical constructs, directly enhancing patients’ ability to navigate post-discharge demands. Finally, to operationalize these strategies, specific recommendations for multidisciplinary discharge planning are essential: collaboration among oncologists, nurses, rehabilitation specialists, social workers, and psychologists ensures that assessments are comprehensive, educational content is holistic, and personalized support is seamlessly integrated. Collectively, these approaches translate the study’s findings into tangible practice, with the potential to improve long-term clinical outcomes and enhance quality of life by ensuring discharge education is precisely aligned with the needs of lung cancer patients and equips them to thrive post-surgery.

### 4.2. Limitations and Future Direction

This study has the following limitations that need to be considered: Firstly, the limitation of causal inference of cross-sectional design is that data collection was limited to a single point in time, and it was not possible to clarify the temporal relationship between variables. Second, the extrapolation limitation of the single-center sample, where all samples came from a single tertiary hospital, may lead to selection bias; In addition, tertiary hospitals often have better discharge guidance resources than primary care organizations, which may overestimate the impact of discharge teaching quality on post-discharge coping difficulty. Future research could be developed in the following directions: Firstly, adopting a mixed-methods approach that integrates qualitative interviews with quantitative analyses is recommended to gain in-depth insights into patients’ individualized needs for discharge teaching. Secondly, conducting multicenter studies that include patient samples from hospitals of varying tiers would enable comparisons of disparities in healthcare resources and their influence on post-discharge coping difficulties. Thirdly, a longitudinal study could be implemented to clarify the pathway of the effect of discharge teaching on long-term rehabilitation outcomes.

## 5. Conclusions

This study identified the current status of post-discharge coping difficulty and the mechanisms of relevant factors in postoperative lung cancer patients. It was found that post-discharge coping difficulty among patients was moderate, with the living management dimension being more prominent. Age, marital status, educational degree, monthly household income, and TNM staging were all influential factors. Self-efficacy and discharge readiness in postoperative lung cancer patients act as a chain-like mediator between post-discharge coping difficulties and the quality of discharge education. These findings provide important knowledge for developing targeted interventions and improving patients’ quality of life.

## Figures and Tables

**Table 1:** Characteristics and univariate analysis of PDCDS in participants (*n* = 358).

Variables	Groups	N (%)	Mean±SD	*t*/*F*	*p*
Sex	Female	184 (51.4)	33.65 ± 10.27	0.708	0.401
	Male	174 (48.6)	35.03 ± 9.69		
Age	18–40	35 (9.8)	25.86 ± 9.63	19.018	<0.001
	41–65	223 (62.3)	34.27 ± 9.40		
	≥66	100 (27.9)	37.40 ± 9.79		
Marital status	Unmarried	15 (4.2)	26.73 ± 10.16	6.243	0.002
	Married	310 (86.6)	34.35 ± 9.82		
	Divorced or widowed	33 (9.2)	37.58 ± 10.05		
Educational degree	Primary or below	134 (37.4)	36.93 ± 9.33	13.486	<0.001
	Junior or senior high school	172 (48.0)	33.97 ± 9.78		
	College or above	52 (14.5)	28.79 ± 10.15		
Employment	Employed	204 (57.0)	33.64 ± 10.70	1.354	0.259
	Retired	80 (22.3)	35.78 ± 10.03		
	Unemployed	74 (20.7)	34.64 ± 7.64		
Living alone	Yes	59 (16.5)	35.54 ± 10.54	1.342	0.247
	No	299 (83.5)	34.08 ± 9.89		
Monthly household income	≤CNY 2000 (a)	15 (4.2)	39.73 ± 11.05	3.248	0.022
	CNY 2001–4000 (b)	116 (32.4)	35.53 ± 9.51		
	CNY 4001–6000 (c)	114 (31.8)	34.12 ± 9.81		
	≥CNY 6001 (d)	113 (31.6)	32.58 ± 10.25		
Type of medical insurance	URRBMI	227 (63.4)	34.86 ± 9.92	0.639	0.424
	UEBMI	131 (36.6)	33.40 ± 10.12		
Primary caregiver	Family caregiver	314 (87.7)	33.87 ± 9.83	0.957	0.329
	Care worker or without a caregiver	44 (12.3)	37.55 ± 10.66		
Postoperative length of stay	≤5 days	254 (70.9)	33.59 ± 9.90	0.037	0.849
	≥6 days	104 (29.1)	36.13 ± 10.08		
Pain score at discharge	0	162 (45.3)	34.01 ± 10.54	0.195	0.823
	1	91 (25.4)	34.35 ± 9.99		
	2	105 (29.3)	34.79 ± 9.21		
TNM staging	I	112 (31.3)	28.92 ± 9.78	46.104	<0.001
	II	172 (48.0)	34.65 ± 8.44		
	III	74 (20.7)	41.74 ± 8.73		
Occurrence of pre-discharge complications	Yes	29 (8.1)	34.72 ± 10.01	0.000	0.991
	No	329 (91.9)	34.29 ± 10.02		

Abbreviations: URRBMI, Urban and Rural Residents Basic Medical Insurance; UEBMI, Urban Employee Basic Medical Insurance.

**Table 2:** Scores for each item of the PDCDS (*n* = 358).

Item	Average Score (Mean±SD)	Affiliated Dimension
1. How would you describe your current level of life stress?	4.48 ± 1.51	emotional management
2. What difficulties have you encountered during your recovery process?	4.59 ± 1.62	emotional management
3. What challenges have you faced while taking care of yourself?	4.87 ± 1.76	living management
4. What obstacles have you experienced in the treatment of your illness? (For example: financial issues, choices in medical care, effectiveness of recovery, etc.)	4.82 ± 1.66	living management
5. What difficulties have your relatives or other close individuals felt during your discharge from the hospital?	4.94 ± 1.62	living management
6. To what extent do you require assistance to take care of yourself adequately?	4.92 ± 1.78	living management
7. What level of emotional support do you need during this period of your discharge from the hospital?	5.71 ± 1.91	living management

**Table 3:** Correlations between PDCDS, QDTS, GSES, and RHDS (*n* = 358).

	QDT	QDT1	QDT2	QDT3	GSE	RHD	RHD1	RHD2	RHD3	PDCD	PDCD1	PDCD2
**QDT**	1											
**QDT1**	0.865 **	1										
**QDT2**	0.886 **	0.724 **	1									
**QDT3**	0.878 **	0.601 **	0.690 **	1								
**GSE**	0.399 **	0.285 **	0.323 **	0.429 **	1							
**RHD**	0.568 **	0.448 **	0.482 **	0.564 **	0.523 **	1						
**RHD1**	0.556 **	0.438 **	0.485 **	0.537 **	0.498 **	0.863 **	1					
**RHD2**	0.459 **	0.364 **	0.392 **	0.462 **	0.475 **	0.904 **	0.697 **	1				
**RHD3**	0.523 **	0.414 **	0.429 **	0.526 **	0.442 **	0.857 **	0.640 **	0.661 **	1			
**PDCD**	−0.447 **	−0.349 **	−0.392 **	−0.433 **	−0.575 **	−0.522 **	−0.474 **	−0.475 **	−0.469 **	1		
**PDCD1**	−0.379 **	−0.303 **	−0.343 **	−0.363 **	−0.477 **	−0.474 **	−0.423 **	−0.437 **	−0.426 **	0.906 **	1	
**PDCD2**	−0.446 **	−0.348 **	−0.386 **	−0.434 **	−0.581 **	−0.514 **	−0.470 **	−0.468 **	−0.460 **	0.985 **	0.822 **	1

Abbreviations: QDT, Quality of Discharge Teaching Scale; QDT1, patients’ self-perceived pre-discharge needs dimension; QDT2, actual support received dimension; QDT3, effectiveness and technique of discharge teaching dimension. GSE, General Self-Efficacy Scale. RHD, Readiness for Hospital Discharge Scale; RHD1, personal status dimension; RHD2, coping ability dimension; RHD3, expected support dimension. PDCD, Post-Discharge Coping Difficulty Scale; PDCD1, emotional management dimension; PDCD2, living management dimension. ** *p* < 0.01.

**Table 4:** Effect values of variables in the mediation model.

	*β*	*SE*	Bias-Corrected95%		*p*
			Lower	Upper	
Standardized Direct Effect					
QDTS→PDCDS	−0.154	0.076	−0.307	−0.001	<0.05
QDTS→GSES	0.420	0.051	0.315	0.516	<0.001
QDTS→RHDS	0.520	0.058	0.406	0.631	<0.001
GSES→RHDS	0.361	0.054	0.248	0.460	<0.001
GSES→PDCDS	−0.356	0.051	−0.449	−0.246	0.001
RHDS→PDCDS	−0.264	0.084	−0.434	−0.102	<0.05
Standardized Indirect Effect					
QDTS→GSES→PDCDS	−0.150	0.028	−0.208	−0.098	<0.001
QDTS→RHDS→PDCDS	−0.137	0.048	−0.252	−0.057	0.001
QDTS→GSES→RHDS→PDCDS	−0.040	0.014	−0.073	−0.017	0.001
Standardized Total indirect effect	−0.327	0.060	−0.469	−0.226	<0.001
Standardized Total Effect	−0.481	0.049	−0.574	−0.382	<0.001

Abbreviations: QDTS, Quality of Discharge Teaching Scale. GSES, General Self-Efficacy Scale. RHDS, Readiness for Hospital Discharge Scale. PDCDS, Post-Discharge Coping Difficulty Scale.

## Data Availability

The data supporting this study’s results are available upon request from the corresponding author. However, these data are not publicly available due to privacy or ethical constraints.

## Supplementary Materials

The following supporting information can be downloaded at: https://www.mdpi.com/article/10.3390/curroncol32080468/s1, STROBE Statement—Checklist of items that should be included in reports of cross-sectional studies.
